# Knockdown of Ice-Binding Proteins in *Brachypodium distachyon* Demonstrates Their Role in Freeze Protection

**DOI:** 10.1371/journal.pone.0167941

**Published:** 2016-12-13

**Authors:** Melissa Bredow, Barbara Vanderbeld, Virginia K. Walker

**Affiliations:** 1 Department of Biology, Queen’s University, Kingston, ON, Canada; 2 Department of Biomedical and Molecular Sciences, and School of Environmental Studies, Queen’s University, Kingston, ON, Canada; CSMCRI, INDIA

## Abstract

Sub-zero temperatures pose a major threat to the survival of cold-climate perennials. Some of these freeze-tolerant plants produce ice-binding proteins (IBPs) that offer frost protection by restricting ice crystal growth and preventing expansion-induced lysis of the plasma membranes. Despite the extensive *in vitro* characterization of such proteins, the importance of IBPs in the freezing stress response has not been investigated. Using the freeze-tolerant grass and model crop, *Brachypodium distachyon*, we characterized putative IBPs (*Bd*IRIs) and generated the first ‘IBP-knockdowns’. Seven IBP sequences were identified and expressed in *Escherichia coli*, with all of the recombinant proteins demonstrating moderate to high levels of ice-recrystallization inhibition (IRI) activity, low levels of thermal hysteresis (TH) activity (0.03−0.09°C at 1 mg/mL) and apparent adsorption to ice primary prism planes. Following plant cold acclimation, IBPs purified from wild-type *B*. *distachyon* cell lysates similarly showed high levels of IRI activity, hexagonal ice-shaping, and low levels of TH activity (0.15°C at 0.5 mg/mL total protein). The transfer of a microRNA construct to wild-type plants resulted in the attenuation of IBP activity. The resulting knockdown mutant plants had reduced ability to restrict ice-crystal growth and a 63% reduction in TH activity. Additionally, all transgenic lines were significantly more vulnerable to electrolyte leakage after freezing to −10°C, showing a 13−22% increase in released ions compared to wild-type. IBP-knockdown lines also demonstrated a significant decrease in viability following freezing to −8°C, with some lines showing only two-thirds the survival seen in control lines. These results underscore the vital role IBPs play in the development of a freeze-tolerant phenotype and suggests that expression of these proteins in frost-susceptible plants could be valuable for the production of more winter-hardy crops.

## Introduction

Low temperatures pose a major threat to the survival of overwintering plants. Uncontrolled growth of ice crystals in the apoplast presumably sequesters water from intracellular compartments leading to cellular dehydration, loss of cell membrane integrity, physical rupture of plasma membranes and ultimately death [1]. Special adaptations allow certain plants to withstand freezing and prevent this cascade of damage. Such plants are termed “freeze-tolerant” and have a number of biochemical, metabolic and physiological mechanisms that help prevent cell death at sub-zero temperatures.

Induction of the freezing stress response is facilitated by exposure to low temperatures, a process known as cold acclimation. Since a crucial site of freeze injury is the plasma membrane, changes in composition are important for providing cryostability during freeze-thaw cycles. Although membrane modifications are species specific, they are commonly associated with an increase in phospholipid and unsaturated fatty acid content [2]. In some plants, the accumulation of compatible solutes that act as osmolytes, such as proline and glycine betaine, is reported to protect cells by preventing cellular dehydration [3]. Additionally, changes in gene expression can result in the upregulation of a number of highly-specialized proteins including cold-regulated (COR) proteins [1], cold-shock domain (CSD) proteins [4], and ice recrystallization inhibition proteins (IRIPs) [5], which are also known as antifreeze proteins (AFPs) and ice-binding proteins (IBPs).

IBPs have been identified in a number of freeze-tolerant plant species including the grasses *Triticum aestivum* [6], *Secale cereal* [7], and *Lolium perenne* [8], as well as dicotyledonous plants such as the carrot *Daucus carota* [9]. These proteins have two well-documented properties: ice-recrystallization inhibition (IRI), which restricts ice crystal growth at temperatures close to melting, and thermal hysteresis (TH), or lowering of the freezing point in relation to the equilibrium melting point [10]. Both properties are derived from the ability of IBPs to irreversibly adsorb to ice crystals, modifying their growth [11].

As plant IBPs have low TH activities (measured in fractions of a degree) compared to those observed for IBPs from other organisms (*e*.*g*. 5.5°C in the yellow mealworm beetle [12]), it has long been assumed that the primary function of most plant IBPs is the restriction of ice crystal growth [13–16]. Typically, IBPs are secreted to the apoplast where they can bind to embryonic ice crystals and prevent their growth into larger, more damaging extracellular ice crystals. However, it has been suggested that some IBPs may remain inside cells to offer intracellular protection from freezing damage [17]. The regulation of these proteins *in planta* is an understudied area of inquiry but may involve post-translational modifications (PTMs), including N-linked glycosylation [18] and cleavage of the ice-binding domain from the full-length protein [5].

The growing body of evidence suggests that IBPs play a vital role in the freezing stress response in plants, as well in other species with such proteins. In plants exposed to low temperatures, IBP abundance increases in leaf and vascular tissues, as well as in the roots and crowns of some plants [13,16,19]. Transgenic studies have shown the capacity of plant IBPs, as well as IBPs produced by insects and fish, to elicit a freeze-tolerant phenotype to less hardy plants, as indicated by reduced electrolyte leakage and increased whole plant freezing survival assays [17,20–22]. However, in order to unequivocally demonstrate the crucial role these proteins play in the freezing stress response, an IBP-knockdown must be generated. The multicopy nature of IBP genes in freeze-avoiding metazoans including fish (*e*.*g*. 30–40 copies [23,24]) and insects (*e*.*g*. ~ 17 copies [25]), the paucity of sequence data available for most freeze-tolerant microbe and plant species, as well as inefficient transformation techniques available for some organisms, have hitherto precluded the generation of knockdown lines in many of these species.

Generation of knockdown mutants is possible using the annual temperate grass *Brachypodium distachyon*. This model species is closely related to cereal grain species as well as forage grasses including *L*. *perenne*. *B*. *distachyon* has the ability to acclimate at low non-freezing temperatures and induce a freezing-tolerance stress response that includes cold-regulated induction of IBPs [26,27]. Importantly for the development of an IBP-knockdown, only 7 IBP genes have been identified in *B*. *distachyon* (*BradiIRI* [27]), henceforth termed *Bd*IRIs. Here we verify that all 7 of these proteins function as IBPs through the characterization of both recombinantly-produced proteins and native isoforms. Additionally, because of the molecular tools available for *B*. *distachyon* as well as the relatively low IBP gene copy number, we were able to successfully design a microRNA construct to generate the first IBP-knockdown plants. By comparing the freezing tolerance capabilities of knockdown lines to their wild-type counterparts we have clearly established the protective role of these proteins in freeze-tolerance.

## Materials and Methods

### Bioinformatics Analysis

Putative *Bd*IRI proteins were identified through a BLAST search against the published *B*. *distachyon* genome using the amino acid sequence for the *L*. *perenne* IBP, *Lp*AFP (accession number AJ277399). Amino acids corresponding to putative ice-binding residues were predicted through alignment with the *Lp*AFP amino acid sequence for which the ice-binding residues have been characterized [28] using the ClustalW2 multiple sequence alignment tool (http://www.ebi.ac.uk/Tools/msa/clustalw2/). Potential peptide cleavage sites were predicted by comparing sequences with *Lp*AFP, which is hypothesized to be a processed isoform, and also by using the ExPASy Peptide Cutter (http://web.expasy.org/peptide_cutter/). The SignalP4.1 server (http://www.cbs.dtu.dk/services/SignalP/) was used to identify putative amino (N)-terminal signal peptides for secretion to the apoplast. Protein modeling was done using the Phyre2.0 server (http://www.sbg.bio.ic.ac.uk/phyre2/html/page.cgi?id=index). Possible sites for glycosylation were predicted using the NetNGlyc 1.0 Server (http://www.cbs.dtu.dk/services/NetNGlyc/) for the identification of N-glycosylated sites.

### Plant material and growth conditions

*B*. *distachyon* seeds (ecotype Bd21) were surface sterilized and exposed to a one week stratification period at 4°C (no light) as previously described [29]. The seeds were then sown to soil and grown in a temperature controlled growth chamber (Queen’s University, Kingston, Ontario, CA) for three weeks on a 20 h/4 h light/dark cycle at 24°C/ 18°C, with humidity and light regulated at 70% and 150 μmol m^−2^ s^−1^, respectively. Prior to experimentation, plants were cold acclimated at 4°C on a short day cycle (6 h light) for 2 d, unless stated otherwise.

### Ice-affinity purification of native IBPs

In order to purify native proteins, ice-affinity purification was conducted using three-week-old cold acclimated *B*. *distachyon*. Leaf tissue (20 g) was ground in liquid nitrogen using a mortar and pestle and the resulting powder was added to 20 mL of native protein extraction (NPE) buffer (10 mM Tris-HCl (pH 7.5), 25 mM NaCl) with two EDTA-free protease inhibitor tablets (Roche). After gentle overnight shaking at 4°C, cellular debris was removed by sieving the lysate twice through three layers of cheesecloth with the remaining debris pelleted by centrifugation at 4,000 x g for 40 min (4°C). The total soluble lysate was used for ice-affinity purification using a modified protocol [30]. The temperature of the ice probe was lowered to −3°C at a rate of 1°C/ day. The ice fraction was melted and the recovered proteins were concentrated by centrifugation (~2 mg/ L). Protein concentration was determined using the Pierce BCA Protein Assay Kit (Fisher Scientific).

A sample of the concentrated protein (2 μg) was sent to the SPARC BioCentre at the Hospital for Sick Children (Toronto, ON CA) for liquid chromatography mass spectrometry (LC-MS/MS). Proteins were subjected to an in-solution tryptic digestion using bovine trypsin and subsequently purified with ZipTips Pipette Tips (Millipore) prior to mass spectrometry using Q Exactive (ThermoFisher) instrumentation. Data were analyzed using PEAKS studio and predicted peptides were aligned against the *B*. *distachyon* NCBI database. Searches for PTMs, relative abundance and other properties were conducted using Scaffold Proteome Software (http://www.proteomesoftware.com/products/free-viewer/).

### Crude cell and apoplast extracts

For the analysis of ice-binding activity of wild-type and transgenic lines, cell extracts were prepared by grinding leaves, which had been frozen with liquid nitrogen, using a mortar and pestle and suspending the resultant powder in NPE buffer. After shaking the suspension for 18 h at 4°C, the samples were centrifuged and the soluble fraction was collected for analysis, as described for the ice-affinity purification protocol. Apoplast extracts were prepared using a protocol previously described by Fursova and colleagues (2009) [31]. Briefly, ~0.2 g of cold acclimated leaf tissue was placed in a syringe containing pre-chilled extraction buffer (25 mM Tris, 50 mM EDTA, and 150 mM MgCl_2_; pH 7.4) and put at 4°C under vacuum for 30 min. After vacuum infiltration, the leaves were patted dry, centrifuged at 4,000 x g for 25 min (4°C) and the supernatant was used for analysis.

### Cloning and purification of recombinant *Bd*IRI proteins

The open reading frames (ORFs) corresponding to the genome accession numbers Bradi5g27350.1 (*BdIRI1*), Bradi5g22880.1 (*BdIRI2*), Bradi5g27340.1 (*BdIRI3*), Bradi5g27330.1 (*BdIRI4*), Bradi5g27310.1 (*BdIRI5*), Bradi5g27300.1 (*BdIRI6*), and Bradi5g22870.1 (*BdIRI7*) were synthesized by GeneArt™ (Invitrogen, Carlsbad, CA, USA). The nucleotides coding for the stop codon were removed in order to incorporate a 6 residue histidine (His)-encoding tag for each of these genes. The ORFs were then ligated into pET24a(+) vectors and transformed into Origami2™(DE3) competent cells designed to fold proteins that require disulphide bridge formation. Sequences were confirmed for all constructs after each cloning step (Plateforme de séquençage et de génotypage des genomes; Québec City, QC, CA).

Bacterial cultures were grown to an optical density (OD) of 0.8 at a wavelength of 595 nm and induced with 0.5 mM of isopropyl β-D-1-thiogalactopyranoside (IPTG) at 18°C for 48 h. Induced cells were lysed using a French press (ThermoFisher Scientific, Nepean, ON, CA) and the soluble protein homogenates were collected before the His-tagged proteins were subsequently purified using a Nickel-NTA agarose column (Qiagen, Toronto, ON, CA). Purified protein was dialyzed against buffer containing 20 mM Tris HCl (pH 8.0), 100 mM NaCl and 10% glycerol at 4°C for 32 h.

### MicroRNA design and construction

The artificial microRNA for attenuation of putative *B*. *distachyon* IBP expression was designed using the WMD3 Web MicroRNA Designer (http://wmd3.weigelworld.org/cgi-bin/webapp.cgi) to target the expression of all 7 isoforms (S1A Table). The construct was assembled according to the cloning protocol described for the design of artificial microRNAs (amiRNAs) (http://wmd3.weigelworld.org/downloads/Cloning_of_artificial_microRNAs.pdf). The amiRNA containing the precursor miR319a in a pRS300 plasmid (Addgene) was used as a template for site-directed mutagenesis. Fragments “a” and “b” and “c” were generated through PCR using Pfu DNA polymerase (Thermo Fisher Scientific) using the following conditions: 94°C for 5 min, followed by 40 cycles of 94°C (30 sec), 52°C (30 sec) and 72°C (2 min) and a final elongation of 72°C for 5 min. Fragment “d” was generated using the same PCR protocol described above with fragments “a”, “b”, and “c”. Fragment “d” was digested with PmlI and BglII restriction enzymes and ligated into the binary vector pCambia1305.1 (pCambia1305.1:mirBdIRI). Prior to transformation the completed sequence was confirmed by sequencing as described for other constructs. We verified that the amiRNA sequence (TAGGTTGAGCGACTCCCACTG) would not attenuate the expression of other unintended sequences using the Target Search provided by the WMD3 website (http://wmd3.weigelworld.org/cgi-bin/webapp.cgi?page=TargetSearch;project=stdwmd), allowing for 5 mismatches.

### Generation of transgenic *B*. *distachyon* plants

The pCambia1305.1:miRBdIRI construct was transformed into *Agrobacterium* strain AGL1 (Invitrogen) using electroporation and subsequently into *B*. *distachyon* using an established protocol for *Agrobacterium*-mediated transformation [32]. Transgenic plants were selected on 0.5x Murashige and Skoog (MS) medium plates containing hygromycin (50 μg/mL). Four independent lines were generated and designated miRBdIRIa-mirBdIRId. Similarly, pCambia1305.1:mOrange (UniProtkB ID DOVWW2_DISSP) lines were generated.

### *B*. *distachyon* IBP transcript analysis

RNA was collected from the leaves of cold acclimated plants using the RNeasy Plant Mini Kit (Qiagen, Toronto, ON, CA) and used for cDNA synthesis using Superscript® III First-Strand Synthesis System (Invitrogen, Carlsbad, CA, USA). PCR was performed using isoform-specific primers (S1A Table) under the following program: 95°C for 5 min, followed by 40 cycles of 94°C (30 sec), 53°C (30 sec), and 72°C (30 sec) and a final elongation of 72°C for 1 min. Amplification of a ‘housekeeping gene’, s-adenosylmethionine decarboxylase transcript (*SamDC*) [33], used as a gel loading reference for IBP transcripts, was done using the same program but with a 55°C annealing temperature.

### Ice-binding and protein activity assays

Prior to analysis of ice-binding activity, protein concentrations for recombinant proteins, as well as cell extracts were determined as indicated earlier. IRI activity, ice-shaping and TH measurements were assessed using protocols that have been optimized for the analysis of ice-binding activity of plant proteins [30]. All experiments were done in triplicate.

### Electrolyte leakage assay

In order to assess the level of membrane damage associated with freezing, three-week-old cold acclimated plants were used to conduct electrolyte leakage assays using a modified protocol [20]. Briefly, one leaf was cut from the base of each plant, placed in 100 μL of deionized water, and immersed in a programmable circulating ethylene glycol bath set to 0°C. After lowering the temperature to −1°C over 30 min, the sample was nucleated with a single ice chip to initiate ice crystal growth. The temperature of the bath was then lowered by 1°C/ 15 min to a final temperature of −10°C and samples were allowed to recover overnight at 4°C. Leaves taken from identically treated cold acclimated plants that remained at 4°C served as controls. All samples were transferred into conical tubes containing 25 mL of deionized water, and shaken at 150 rpm for 18 h. Conductivity measurements were taken before (C_i_) and after (C_f_) autoclaving samples to account for the total leaf mass, using a direct reading conductivity meter (Bach-Simpson Ltd., London, ON, CA) and presented as a percentage (100 C_i_C_f_^−1^) with 10 individual plants for each independent line. The entire procedure was carried out in triplicate.

### Whole plant survival assay

Three-week-old plants sown to soil and exposed to a 2 day cold acclimation period, were placed in a temperature regulated growth chamber set to −1°C and allowed to acclimate for 12 h. Plants were sprayed lightly with ice water and incubated at −1°C for 1 h before the temperature was then lowered to −8°C (1°C/h). Plants recovered at 4°C (no light) for 2 days prior to returning to standard growth conditions for 7 days, after which the percent survival was recorded. Assays were done using 10 plants per independent line and the entire procedure was repeated in triplicate.

## Results

### Bioinformatics analysis

**Fig 1:**
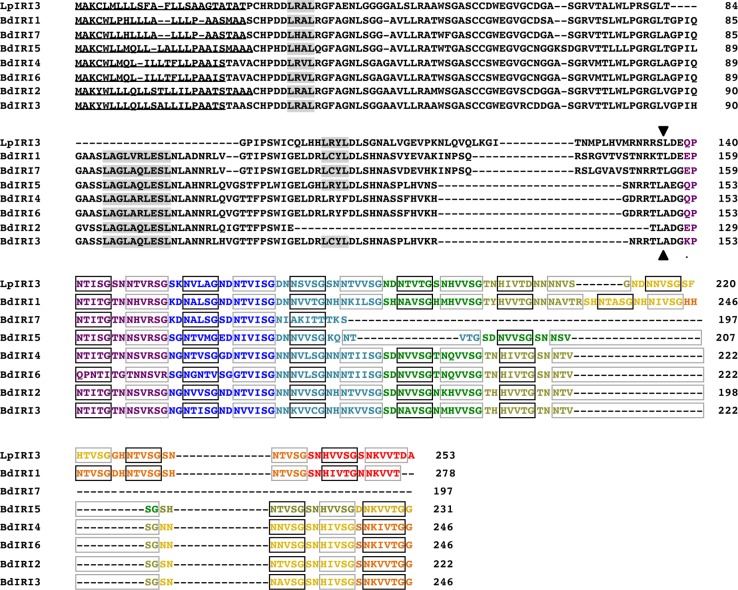
Amino acid sequence alignment of *Bd*IRI proteins. The *Bd*IRI amino acid sequences were aligned against the sequences of three *L*. *perenne* IRI isoforms: *Lp*IRI4 (EU680851), *Lp*IRI1 (EU680848), and *Lp*IRI3 (EU680850), using ClustalW2 multiple sequence alignment tool. N-terminal signal peptides, predicted by the SignalP 4.1 server, are underlined. Conserved leucine-rich repeat motifs (LXXL, where x represents a non-conserved residue) are highlighted in grey. The ice-binding motifs NXVXG and NXVXXG are outlined in black and grey boxes, respectively. The amino acids predicted to compose one β-helical turn, as indicated by the Phyre2 algorithm are presented as different colors. A black arrow marks the location of potential cleavage sites in the aligned residues (see text). Individual columns of residues are annotated as: (*) denoting a single, fully conserved reside; (:) denoting conservation between groups of highly similar properties (scoring > 0.5 in the Gonnet PAM 250 score), and (.) denoting conservation between groups of weakly similar properties (scoring = <0.5 in the Gonnet PAM 250 score).

Putative *Bd*IRI proteins were identified through a BLAST search using the amino acid sequence corresponding to the *Lp*AFP gene sequence (genome accession number AJ277399). Seven sequences were identified in the *B*. *distachyon* database corresponding to accession numbers Bradi5g27350.1, Bradi5g22880.1, Bradi5g27340.1, Bradi27330.1, Bradi5g27310.1, Bradi5g27300.1, Brad5g22870.1, and for ease of nomenclature, these accessions have been designated to encode proteins *Bd*IRI1-7, respectively. *Bd*IRI1-7 show 88%, 84%, 83%, 81%, 77%, 72%, and 57% similarity (67%, 53%, 49%, 47%, 45%, 30%, 21% identity) to *Lp*AFP, respectively. Alignment of putative *Bd*IRI proteins with the amino acid sequences of IBPs from *L*. *perenne* (*Lp*IRI1, *Lp*IRI3, and *Lp*IRI4; EU680848.1, EU680850.1 and EU680851.1) identified conserved motifs found in many plant IBPs. The amino (N)-terminal domain of all the *B*. *distachyon* proteins were predicted to have secretion signals for apoplastic localization followed by a varying number of leucine-rich repeats (LRRs) and a carboxyl (C)-terminal domain with a number of conserved ice-binding motifs (NXVXG/NXVXXG, where X represents an outward facing residue) (Fig 1).

**Fig 2:**
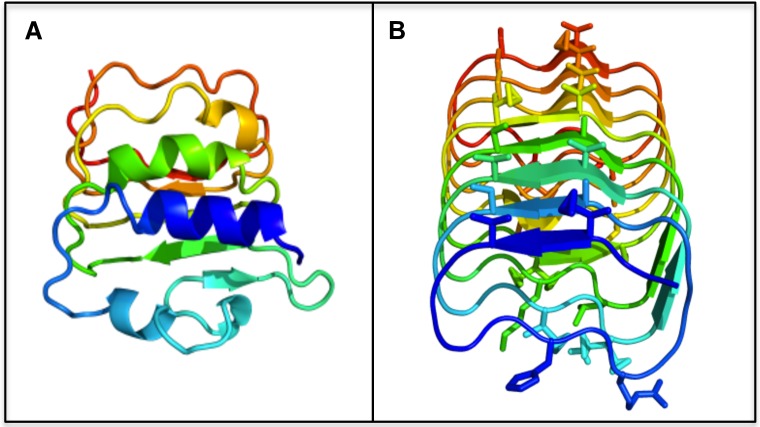
Structural model of the *Bd*IRI1 protein sequence. The amino(N)- (A) and carboxyl(C)- (B) terminal domains were modeled individually using the Phyre2 algorithm. The N-terminal domain modeled to *A*. *thaliana* FLS2 (At5g46330.1) crystal structure corresponding to a flagellin receptor protein. The C-terminal domain was modeled to the crystal structure of *Lp*AFP (AJ277399), an IBP identified in *L*. *perenne*. Putative ice-binding residues identified on the flat surfaces of the C-terminal domain are shown as sticks.

*In silico* modelling (Fig 2A) showed that the N-terminal domains were similar to *Arabidopsis thaliana* FLS2 (an LRR receptor-like kinase) with a highly conserved structure that is shared generally amongst LRR-containing proteins. This consists of loops with β-sheets on one side and irregular α-helices on the other, creating a concave shape on one surface. Similar to other plant IBPs, the C-terminal domain of all 7 proteins were predicted to fold into a right handed β-helix with two flat faces (Fig 2B). Notably, with the exception of Bradi5g27350.1 (*Bd*IRI1), these isoforms contain fewer β-helical turns than IBPs found in other plants, with Bradi5g22870.1 (*Bd*IRI7) only predicted to have two turns.

Putative N-linked glycosylation sites were identified for all 7 isoforms (S2 Table) although it should be noted that such algorithms were developed for animal sequences and thus may not be applicable to plants. Nevertheless, such glycosylation sites were predicted to occur in the N-terminal domain of *Bd*IRI isoforms 1, 2, 3, 4, and 7 and both the N- and C-terminal domain of *Bd*IRI isoforms 5 and 6.

### Identification and characterization of native *Bd*IRI proteins

**Fig 3:**
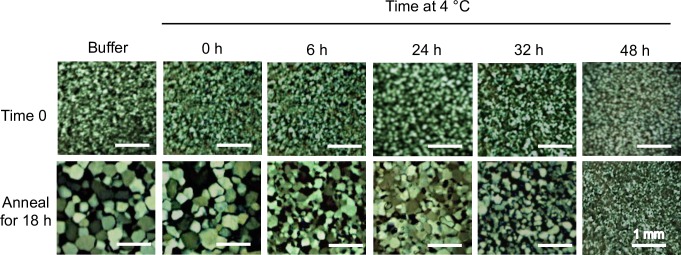
Assessment of ice-binding activity induction in wild-type *B*. *distachyon*. Crude lysates (0.1 mg/mL of total protein) collected from the leaves of *B*. *distachyon* were used to test for IRI activity over a 48 h cold acclimation period at 4°C. Ice crystals were observed at time zero and after annealing at −4°C for 18 h. Assays were done in triplicate.

**Fig 4:**
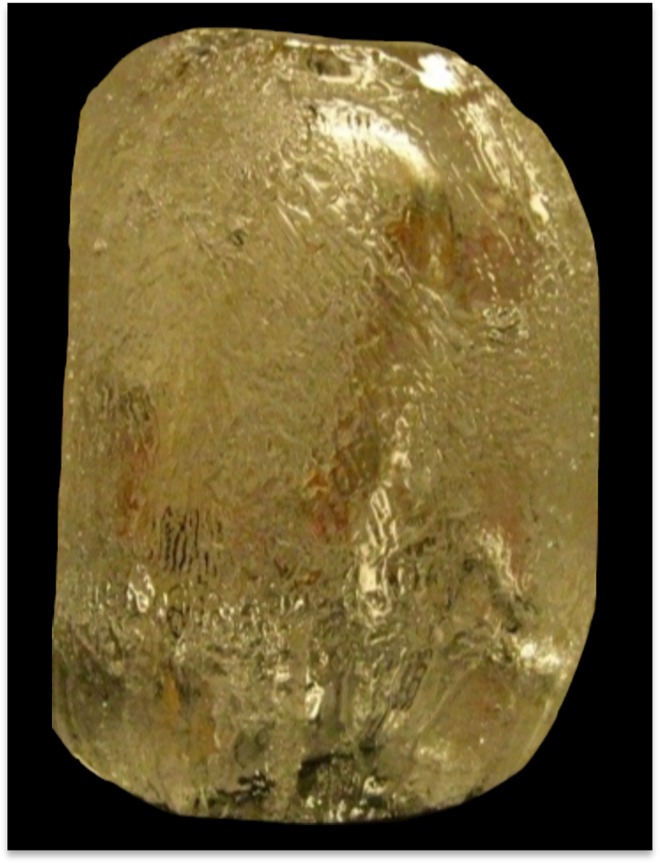
Representative ice hemisphere obtained during the ice-affinity purification of native *Bd*IRI proteins. The polycrystalline ice hemisphere shown was obtained after two rounds of ice-affinity purification using crude cell lysates of cold acclimated (1 week at 4°C) wild-type *B*. *distachyon* leaf tissue (20 g). The ice etching observed across the surface of the ice hemisphere indicates successful incorporation of IBPs. The procedure was conducted in triplicate.

**Fig 5:**
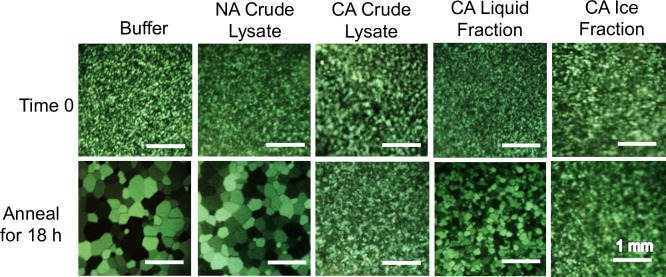
Monitoring ice-binding activity during ice-affinity purification of proteins from *B*. *distachyon* leaves. The crude cell lysates (0.1 mg/mL of total protein) of non-acclimated (NA) and cold-acclimated (CA) (1 week at 4°C) leaf tissue, as well as samples collected from the ice and liquid fractions during the purification procedure were tested for ice-recrystallization inhibition activity. Samples were observed at time 0 and following an 18 h incubation period at −4°C with all assays conducted in triplicate.

**Fig 6:**
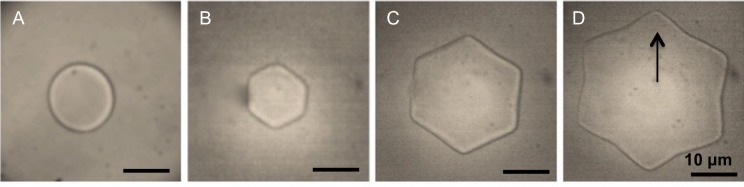
Ice crystal shaping of wild-type *B*. *distachyon* lysates. The morphology of ice crystals formed in the presence of lysates of non-acclimated *B*. *distachyon* (A) was compared to those produced by cold-acclimated (CA) *B*. *distachyon* lysates (B). The ice crystal “burst” observed in the presence of CA lysates is shown in panels (C) and (D). The arrow indicates the direction of the a-axis in one of the images. Assays were conducted using 0.5 mg/mL of total protein, in triplicate.

Restricted ice crystal growth was clearly observed in crude cell extracts collected from wild-type *B*. *distachyon* following a 48 h cold acclimation period, but not following shorter cold treatments (Fig 3). After a 48 h acclimation period, native *Bd*IRI proteins were isolated using ice-affinity purification, which resulted in “ice etching”, observed as a rough and sometimes tinted polycrystalline hemisphere of ice, indicating protein incorporation (Fig 4). The melted ice fraction had high IRI activity (at 0.1 mg/mL protein; Fig 5), with a mean TH activity of 0.15°C ± 0.005°C (0.5 mg/mL). The morphology of the ice crystal was hexagonal, suggestive of binding to the primary prism plane of ice (Fig 6), and when the equilibrium was exceeded, the ice crystal’s ‘burst’ showed growth along the a-axes. This type of burst morphology is typical of samples with low IBP concentrations.

**Fig 7:**
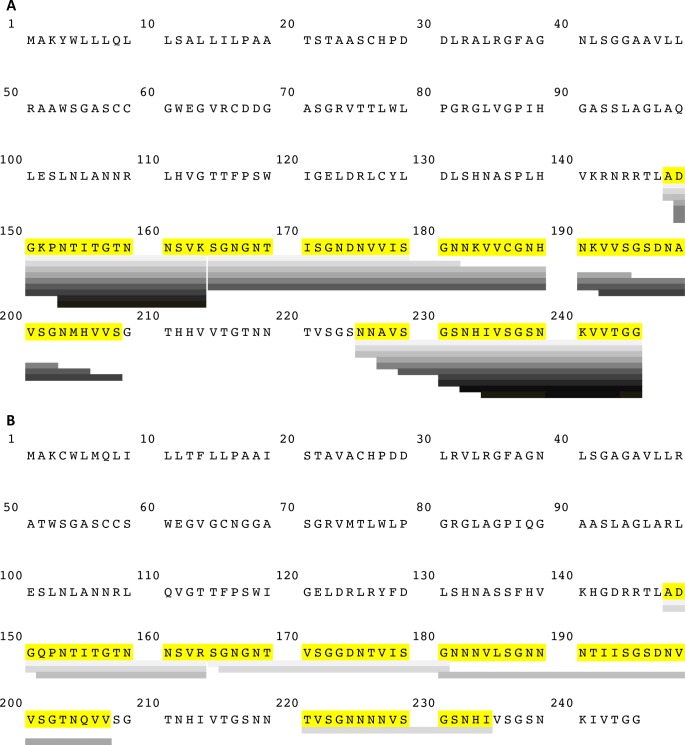
Alignment of *Bd*IRI peptides recovered by ice-affinity purification. Peptide fragments identified by LC-MS/MS were aligned against the full-length amino acid sequences of *Bd*IRI3 (A) and *Bd*IRI4 (B). Amino acid coverage is indicated in yellow. Peptide fragments recovered by LC-MS/MS are indicated by grey boxes.

Two predicted IBP sequences were identified in the ice-purified lysates of cold acclimated *B*. *distachyon* through LC-MS/MS with 100% identity to proteins encoded by genes corresponding to genome accession numbers Bradi5g27340.1 and Bradi5g27330.1 (*Bd*IRI-3 and -4), with the former isoform being 25-fold more abundant (Fig 7). No sequence reads were identified for the LRR domain (corresponding to amino acid positions 1–148 for both B*d*IRI-3 and -4), indicating that the purified proteins were processed, and further that the cleaved N-terminal domain was not recovered by ice-affinity. Use of the ExPASy Peptide Cutter had predicted a number of cleavage sites, however, since it is unlikely that cleavage would arise within the ice-binding domain itself, we had hypothesized that cleavage would occur just after the LRR domain. Cleavage sites were identified 3 amino acids N-terminal to the predicted ice-binding motifs corresponding to asparagine endopeptidase hydrolysis, aligning perfectly with the algorithm-based predictions (Fig 1), and the recovered sequence reads obtained after LC-MS/MS. No other PTMs were identified for either peptide.

### Ice-binding activity of recombinant *Bd*IRI proteins

**Fig 8:**
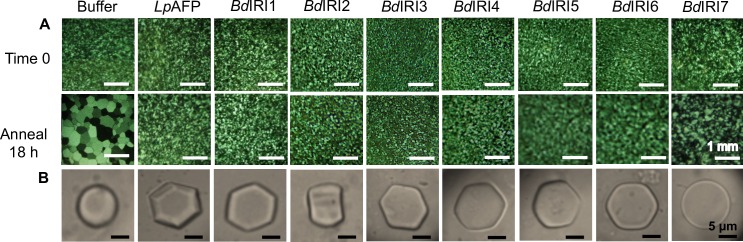
Ice-binding activity of *Bd*IRI proteins produced in *E*. *coli*. Ice recrystallization inhibition activity was observed for all 7 *Bd*IRI recombinant proteins as indicated, as well as purified recombinant *Lp*AFP and buffer controls (see text). Images were captured immediately (time 0) and following an annealing period of 18 h at −4°C (A). Ice crystal morphologies were also observed for all proteins (B). Assays were conducted using 1 mg/mL of recombinant protein, in triplicate with representative photos shown.

Initially, DNA fragments corresponding to the *BdIRI* ORFs were cloned in pET24a(+) vectors and expressed in BL21 cells resulting in the recovery of ~95% of the recombinant protein in the insoluble protein fraction [15]. Various methods including denaturation of the recombinant proteins followed by renaturation *in vitro*, as well as expression in ArcticExpress™ competent cells were tried in an attempt to solubilize the proteins but these procedures all proved unsuccessful. Thus, constructs were made for expression in Origami2™ (DE3) cells, with the result that the recombinantly-produced proteins were mostly soluble (~75%) and all 7 proteins demonstrated IRI activity at 1 mg/mL (Fig 8A). Five of the isoforms were very active, showing no growth of ice crystals when diluted below 0.1 mg/mL, but two isoforms, *Bd*IRI6 and *Bd*IRI7, lost IRI activity at 0.5 mg/mL and 0.1 mg/mL, respectively (S1 Fig). All proteins, except *Bd*IRI*7*, appeared to bind to the primary prism and basal planes of ice (Fig 8B). This was indicated by the hexagonal shaping of individual ice crystals in the presence of *Bd*IRI1 and *Bd*IRI3-6. Ice crystals formed in the presence of *Bd*IRI2, unlike the other *Bd*IRI isoforms which were oriented with the basal plane facing forward, displayed the prism plane (Fig 8B). This “rectangular” shaping is also suggestive of adsorption to the primary prism plane by *Bd*IRI2. Low TH activities were observed for all *Bd*IRI proteins (Table 1A). Compared to other isoforms, *Bd*IRI6 and *Bd*IRI7 had a decreased capacity to adsorb to ice crystals and lower TH activity. This activity was directly correlated with the putative number of ice-binding motifs (Fig 1) as well as the overall percent similarity to *Lp*AFP.

**Table 1:** Thermal hysteresis readings for recombinant proteins (A) and crude cell lysates of plants (B).

**A. Isoform**	**Thermal Hysteresis Activity (°C)**
*Bd*IRI1	0.093 ± 0.002
*Bd*IRI2	0.082 ± 0.002
*Bd*IRI3	0.079 ± 0.002
*Bd*IRI4	0.087 ± 0.004
*Bd*IRI5	0.081 ± 0.003
*Bd*IRI6	0.064 ± 0.004
*Bd*IRI7	0.059 ± 0.003
*Lp*AFP	0.14 ± 0.001
Buffer	0
**B. Line**	**Thermal Hysteresis Activity (°C)**
Wild-type	0.079 ± 0.012
miRBdIRI-a	0.013 ± 0.003
miRBdIRI-b	0.011 ± 0.014
miRBdIRI-c	0.034 ± 0.017
miRBdIRI-d	0.009 ± 0.002

Notes: ± represents the standard deviation of the mean Recombinant proteins were tested at a protein concentration of 1 mg/mL and the lysates of wild-type and knockdown lines (miRBdIRIa-d) were tested at 0.5 mg/mL. All assays were conducted in triplicate.

### Attenuation of IBP expression in transgenic *B*. *distachyon*

**Fig 9:**
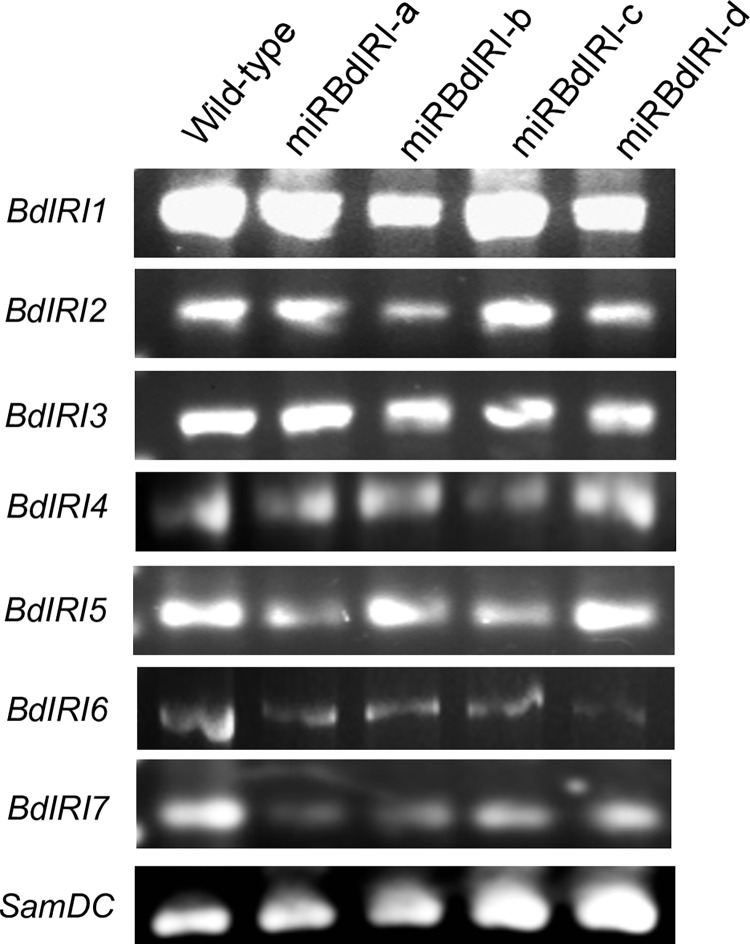
Transcript analysis of *BdIRI*s in wild-type and knockdown (miRBdIRI) plants. Transcripts were amplified for four independent lines (miRBdIRIa-d) as well as wild-type plants with PCR performed using the isoform-specific primers listed in Table 1B. The *SamDC* transcript was used as a PCR loading reference. Assays were done in triplicate.

**Fig 10:**
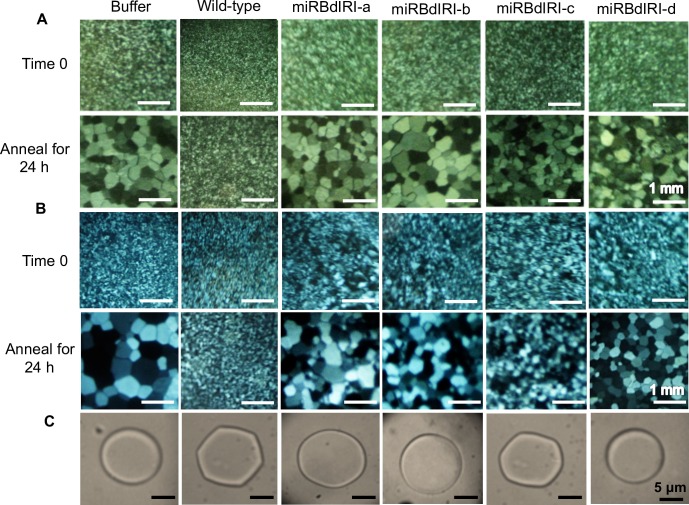
Ice-binding activities in wild-type *B*. *distachyon* and transgenic knockdown lines (miRBdIRIs). Ice-recrystallization inhibition was assayed in buffer controls and crude cell lysates (A) as well as apoplast extracts (B), from wild-type plants and knockdown lines (miRBdIRIa-d) after annealing at −4°C for 24 h, at a total protein concentration of 0.1 mg/mL. Ice crystal morphologies (C) were tested at 0.5 mg/mL of total protein collected from crude cell extracts. All assays were performed in triplicate.

Following cold acclimation, transcripts for all *BdIRI* genes were present in the leaf tissue of both wild-type and miRBdIRI-knockdown lines (Fig 9). No transcripts were present in non-acclimated leaf tissue collected from wild-type plants (S2 Fig). When crude cell lysates and apoplast extracts were collected from cold acclimated knockdown lines, they showed reduced IRI activity (Fig 10A and 10B) compared to wild-type controls. As well, the circular disc morphology of the ice crystals formed in the presence of the cold acclimated knockdown lines was distinct from the hexagonal ice crystals seen in the presence of wild-type lysates (Fig 10C). Accordingly, the average TH activity of the miRBdIRI plants was reduced by 57–88% across all four transgenic lines compared to wild-type plants (Table 1B). Notably, miRBdIRI line-c indicated somewhat weaker translational repression than other lines, nevertheless ice-binding activity was clearly reduced. Analysis of the ice-binding activity of *B*. *distachyon* lines transformed with control pCambia:mOrange constructs confirmed that vector insertion did not result in the altered ice-binding phenotypes.

### Phenotypes of transgenic IBP knockdown plants

Each of the four independent miRBdIRI-knockdown lines were maintained as heterozygotes since homozygosity is not required for attenuation using microRNAs. They were together notable for their distinct phenotype including shorter stature (S3 Fig) and dramatically reduced seed number in each pod compared to wild-type plants (Table 2). Empty vector control plants did not show reduced seed set, with an average of 76–361 seeds across five independent lines, suggesting this phenotype was not associated with IBP attenuation. Indeed, the very low number of viable seeds made the analysis of freeze tolerance in the transgenic plants a challenge.

**Table 2:** Developmental characteristics of wild-type *B*. *distachyon* and miRBdIRI knockdown lines.

	Parameter			
Line	Height (cm)	Number of Florets	Number of Seeds	Percent Germination
Wild-type	29.3 ± 2.3	204.3 ± 27.2	150.7 ± 31.5	89.4 ± 3.6
miRBdIRI-a	18.8 ± 6.5	191.6 ± 40.6	3.9 ± 4.0	41.6 ± 13.7
miRBdIRI-b	23.6 ± 4.8	224.2 ± 21.3	9.1 ± 7.1	62.7 ± 12.4
miRBdIRI-c	17.9 ± 5.0	197.7 ± 46.6	7.9 ± 5.3	70.4 ± 4.6
miRBdIRI-d	12.8 ± 4.8	200.1 ± 16.2	9.4 ± 6.0	77.6 ± 20.7

Notes: ± represents the standard deviation from the mean. Data was compiled from 5 independent growth trials using at least 8 plants for each line (miRBdIRIa-d) and wild type. Plant heights were measured at 12 weeks. Percent germination was determined by plating seeds on MS agar and growing under standard growth conditions (refer to experimental procedures).

**Fig 11:**
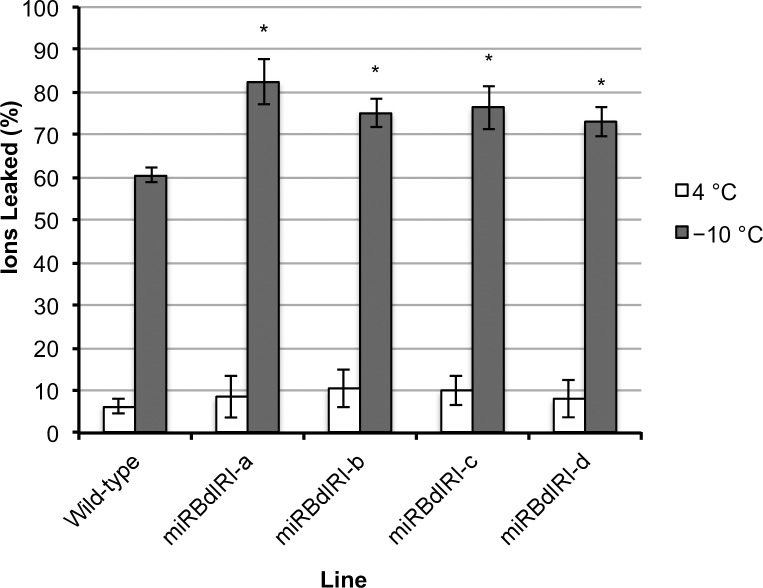
Ion-leakage assays conducted using leaf tissue from cold acclimated wild-type *B*. *distachyon* and miRBdIRI knockdown lines. Controls were kept at 4°C (open bars) and treated samples (dark bars) were frozen to a final temperature of −10°C over a 4 h period before assayed for ion leakage (%). Error bars represent standard error of the mean with significance denoted by an asterisk and indicating p<0.005 (unpaired T-test, one-tailed). Experiments were done in triplicate (n = 10).

It has been hypothesized that IBPs protect plasma membranes through freeze thaw cycles by restricting the growth of extracellular ice crystals, preventing dehydration-induced rupture of the plasma membrane [34], as well as by associating with the plasma membrane, providing stabilization [35–37]. To test these hypotheses, electrolyte leakage was assayed in the four lines. Compared to leaves from cold acclimated wild type plants, a significant increase in electrolyte leakage was seen across all transgenic lines (T-test, p<0.005) following freezing of leaf samples to –10°C, with an overall increase of 21.9%, 14.8%, 15.9% and 12.6% for miRBdIRI knockdown lines a-d, respectively (Fig 11). Few electrolytes were released from plants incubated at 4°C.

**Fig 12:**
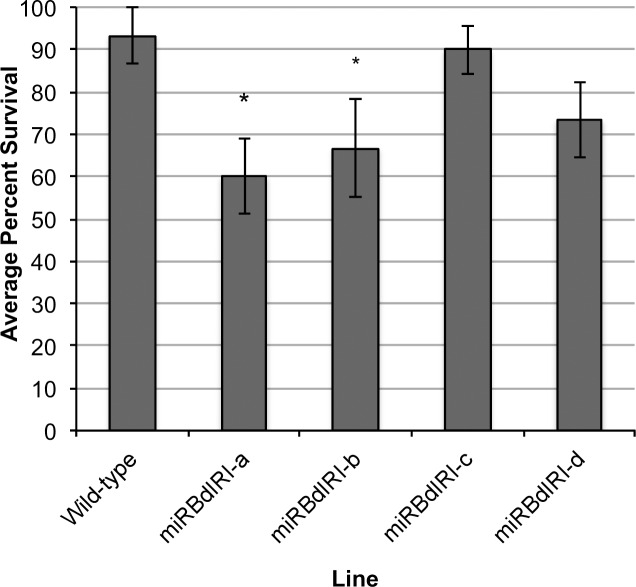
Whole plant freeze survival assays of wild-type *B*. *distachyon* and transgenic knockdown lines (miRBdIRIs). Cold-acclimated wild-type and knockdown lines (miRBdIRIa-d) were slowly frozen to −8°C at a rate of 1°C/ h. Plants were then allowed to recover for 2 days at 4°C (no light), and placed back in standard growth conditions for 7 days, after which survival was recorded. Values are represented as a mean of three trials (n = 10 plants) with error bars representing standard error of the mean, and significance denoted by asterisks for p<0.005 (unpaired T-test, one-tailed).

Whole plant freeze survival assays conducted at −8°C showed 93.3% survival of wild-type plants (Fig 12). In contrast, survival was reduced in miRBdIRI-knockdown lines, with mean survival rates of 60.2%, 66.7%, 89.7%, and 73.3% for miRBdIRI lines a-d lines, respectively. Of note, miRBdIRI-c plants showed the highest level of freeze survival of all independently generated transgenic lines, in accordance with the higher levels of IRI, ice shaping and TH activity when compared to other transgenic lines (Fig 10 and Table 1B). Despite the fact that extraordinary efforts were put into place to cultivate all these lines, there was only sufficient material to test 10 plants per independent transgenic line (repeated in triplicate). A reduction in survival was observed in all lines, however, likely due to the restricted sample size, there was a statistically significant reduction in two of the lines.

## Discussion

The evolution of IBPs in freeze-tolerant organisms points to their role in protecting cells from damage incurred at sub-zero temperatures. However, without a knockdown to verify the role of these proteins, these claims remained formally unsubstantiated. Accordingly, we have identified and functionally characterized 7 IBPs in the model cereal, *B*. *distachyon*, and generated knockdown lines with demonstrably reduced IBP activity. Using these knockdown lines we have now confirmed that IBPs are important players in the freezing stress response. In these plants specifically, and in conjunction with other transgenic expression studies (*e*.*g*. [20, 22]), our results highlight the value of IBPs as candidates for the development of freeze-tolerant crops.

Analysis of the ice-binding activity of wild-type *B*. *distachyon* cell extracts showed that following a short cold acclimation period, these plants induced the expression of proteins capable of restricting ice crystal growth (Fig 3), shaping ice (Fig 6) and depressing the freezing point (0.14°C at 0.5 mg/mL). We isolated proteins that adsorbed to ice in order to validate the identity of native *Bd*IRI proteins. Somewhat surprisingly, only 2 of the 7 *Bd*IRI proteins (*Bd*IRI3 and *Bd*IRI4) were recognized following ice-affinity purification. This may be due to differing capacities for ice crystal adsorption, although this is not obvious from TH, IRI, or ice-shaping activities, except for the less active *Bd*IRI6 and *Bd*IRI7 proteins (Table 1A and Fig 8). Notably, these two isoforms have the least similarity to *Lp*AFP, and the reduced number of conserved ice-binding residues may result in a lower capacity for ice crystal adsorption. However, it is not known why peptides corresponding to the other three proteins were not recovered; we speculate that this could reflect varying isoform expression in leaves at this particular point in the cold acclimation process.

The isolated native *Bd*IRI proteins provided insight into the processing of IBPs *in planta*. Previous research has shown that some plant IBPs undergo PTMs, including N-linked glycosylation [8,19,38,39,40,41]. While glycosylation is necessary for the ice-binding activity of IBPs produced in *Solanum dulcamara* [18], both glycosylated and deglycosylated forms of IBPs produced in other plants have shown similar levels of ice-binding activity [8,19,39]. The identification of algorithm predicted N-linked glycosylation sites in the coding sequences of *Bd*IRI proteins (S2 Table) suggested that these proteins too, may be glycosylated. However, according to LC MS/MS analysis, the two *Bd*IRI isoforms recovered from ice-affinity purification were not glycosylated. IBPs isolated from *T*. *aestivum* are also not glycosylated [42] and while the IBPs produced in *L*. *perenne* are postulated to be glycosylated, experiments with the bacterially-produced recombinant IBP have shown that ice-binding occurs through the protein backbone and glycosylation is not required for activity [8,43]. Together, this evidence suggests that while glycosylation may be an important PTM for the activity of some IBPs, it is likely not required for the activity of those produced in the Pooideae family of freeze-tolerant grasses.

In addition to glycosylation, some studies have suggested that IBPs from freeze-tolerant grasses may undergo specific proteolytic cleavage *in planta* [5]. Indeed, the native *Bd*IRI3 and *Bd*IRI4 proteins appeared to have been fully processed, with no peptides corresponding to the N-terminal LRR domain identified in the ice-purified fraction. The processed proteins match algorithm-based predictions (Fig 1) with hydrolysis occurring three amino acids N-terminal to the ice-binding domain. It should be mentioned that studies with recombinant IBPs that are N-terminally tagged are capable of adsorption to ice (for example, *Lp*AFP [43]). Thus while even non-processed IBPs could adsorb to ice, it may be the case that removal of the LRR domain, which is predicted to have a concave structure on one surface, is required for maximal ice crystal adsorption, since efficiency of adsorption has been associated with the “flatness” of the ice-binding surface [44]. Following processing, the LRR domain itself could have a function, as suggested below.

Assessment of the ice-binding activity of the recombinantly-produced proteins revealed that all isoforms had the capacity to restrict ice crystal growth, depress the freezing point, and with the exception of *Bd*IRI7, shape ice (Table 1A and Fig 8). In addition to the lowest TH activities, ice crystals annealed in the presence of *Bd*IRI6 and *Bd*IRI7 were noticeably larger than those observed with the other five isoforms. However, the other recombinant isoforms with comparatively higher TH activity were still modestly lower (~0.1°C at 1 mg/mL) (Table 1A) than *Lp*AFP (~0.3°C at 1.5 mg/mL) [43] and *Dc*AFP (~0.3°C at 1 mg/mL) [45]. Nonetheless, the ice-binding activity of *Bd*IRI isoforms was substantially greater than purified proteins from winter wheat, which did not show TH or ice crystal shaping at reasonable concentrations (~ 0.33°C at 60 mg/mL) [14]. While TH activity may be the hallmark property of IBPs, it is well documented that IRI activity is more functionally relevant in plants than in other freeze-avoidant species [46]. In this regard, while *Bd*IRI isoforms 1–5 can most certainly be classified as IBPs, the lower IRI activity of *Bd*IRI6 and *Bd*IRI7 at concentrations below 1 mg/mL suggests that if these isoforms were expressed by themselves, they might not serve as effective IBPs *in planta*.

A marked difference in the activity levels of native extracts (0.14°C at 0.5 mg/mL) and recombinantly-produced isoforms (0.06–0.09°C at 1 mg/mL) (Table 1A) was observed. Initially, we hypothesized that glycosylation might be required for the optimal ice-binding activity of these proteins. However, since glycosylation was not seen in the two native IBPs identified after mass spectrometry, the enhanced ice shaping and TH activity seen with native proteins compared to the individual recombinantly-produced *Bd*IRIs could instead be explained by the presence of more than one isoform. Indeed, the expression of multiple IBPs is reported to enhance ice-binding and TH activity [20,47,48]. Additionally, since the recombinant proteins used in this study were those representing the full coding sequence and native *Bd*IRI proteins appear to be cleaved, it is possible that the absence of processing could also explain the discrepancy between ice crystal adsorption in the native and recombinant IBPs.

Having now verified the ice-binding activity of *Bd*IRI proteins, we generated four independent knockdown lines with attenuated IBP activity. The presence of transcript in knockdown lines indicated that the miRNA regulates expression by repressing the translation of the *Bd*IRI transcripts as has been seen with other miRNAs in plants [49–51]. The loss of ice-binding activity in transgenic lines was observed in crude cell extracts containing both intracellular and extracellular components, but also in the apoplastic extracts of IBP knockdowns (Fig 10). Most plant IBPs have been shown to localize to the apoplast [7,14,20,52–53] and recent research has demonstrated that localization of IBPs to the extracellular space may be required for full freeze protection [20]. In addition to the identification of putative extracellular signal peptides in the N-terminal domain of all 7 *Bd*IRIs (Fig 1), the presence of IRI and TH activity, as well as ice shaping in the apoplastic extracts of wild-type *B*. *distachyon* lines, suggests that *Bd*IRI proteins are localized to extracellular compartments.

Electrolyte leakage assays demonstrated that attenuation of IBP expression resulted in a loss of cell integrity at sub-zero temperatures when compared to wild-type *B*. *distachyon* (Fig 11). Since electrolyte leakage is an indicator of the level of cell membrane stability [54], the substantial increase in the percentage of leaked ions in the transgenic lines following freezing indicates an increase in membrane damage associated with the reduction in *Bd*IRI levels. Previous research has suggested that some plant IBPs could be affiliated with membranes, presumably to provide stabilization during freeze-thaw cycles [36,37,55]. However, given the extracellular localization of these proteins, another possible explanation for the membrane protection is that IBPs restrict the growth of ice crystals in the apoplastic space during freezing, preventing physical damage to the membranes caused by the growth of large ice crystals. Additionally, it is thought that plasma membrane association occurs though the sugar moiety of antifreeze glycoproteins (AFGPs) [56] and since *Bd*IRI proteins appear to be not glycosylated, an association with membranes may be unlikely.

Although it has long been speculated that IBPs are required for freezing protection, until now, there has been no direct test of the importance of this particular protein family for low-temperature endurance. Whole plant survival assays (Fig 12) revealed that all IBP knockdown lines had reduced rates of viability following freezing. Although a significant decrease in survival was only seen in two lines, it is noteworthy that viability was correlated with the level of ice-binding activity. Specifically, the knockdown line miRBdIRI-c, which retained the highest IRI and ice-shaping activity of all transgenic lines (Fig 10) also showed the greatest freeze survival. This observation suggests that the level of IBP expression is important factor in determining the degree of freeze tolerance. Notwithstanding the low fecundity of the transgenic lines necessitating the limited number of plants for testing (N = 10), marked differences were still seen in three lines. We submit this is evidence of the importance of IBPs in the freezing-tolerance stress response in *B*. *distachyon*.

As indicated, in all independently derived lines, knockdown of IBPs resulted in pleiotropic phenotypes including lower seed set and germination rates, as well as reduced above ground biomass (Table 2). Algorithm-based amiRNA target predictions, using the fully annotated *B*. *distachyon* genome, did not identify any off-target ‘hits’ and therefore, we suspect there is another explanation for this phenotype. One possibility is that these detrimental effects are directly attributable to the knockdown of the N-terminal LRR domain. LRR proteins have roles in inflorescence architecture, the development of floral meristem cells, floral organ abcission, plant defense and pathogen recognition, as well as microsporogenesis and male sterility [57–64]. IBP transcripts have been identified in the embryos and seeds of closely related species [65,66] and although the ice-binding ß-domain may not stable at standard growth temperatures [43], it is possible that the cleaved LRR domain could still accumulate in the apoplast at some stages of development. These observations, although preliminary, warrant the investigation of these additional phenotypes in future studies to fully understand the possible multifunctional roles of IBPs in plants.

To recapitulate, for the first time, we have generated an IBP knockdown to test the role of IBPs in plant freeze tolerance. The attenuation of expression of 7 proteins with confirmed ice-binding activity resulted in plants that suffered increased membrane damage and had lower rates of survival following freezing. Our results suggest that the freeze protection provided by *Bd*IRI proteins could be due to the restriction of ice crystal growth by these proteins. Given the pleiotropic effects incurred on these knockdown lines, future studies using spatial and/or temporally controlled promoter systems would be valuable in order to elucidate the specific roles of these proteins in plant systems.

## Supporting Information

S1 Fig Ice-recrystallization inhibition analysis of recombinant *Bd*IRI isoforms. A dilution series was conducted on purified *Bd*IRI proteins and ice crystals were observed after annealing at −4°C for 18h. Assay was conducted in triplicate.
(TIF)

S2 Fig Transcript analysis using leaf tissue from non-acclimated wild-type *B. distachyon*. Transcripts were amplified for each *Bd*IRI using sequence specific primers (Table 1). *SamDC* served as a PCR loading reference. Assays were conducted in duplicate with identical results.
(TIF)

S3 Fig Phenotypes of transgenic miRBdIRI plants used in this study. Twelve-week-old transgenic *Bd*IRI knockdown lines show shorter stature and less above ground biomass compared to wild-type *B*. *distachyon* plants.
(TIF)

S1 Table Primer sequences utilized for PCR. The primers used for the generation of the miRBdIRI construct (A) and the forward (FW) and the reverse (RV) primers used for reverse-transcription PCR of *Bd*IRI transcripts (B) are shown. Restriction sites are underlined and melting temperatures (T_M_) are indicated.
(TIF)

S2 Table Prediction of N-linked glycosylation in each *Bd*IRI isoform. Predictions using *Bd*IRI1-7 sequences were made using the NetNGlyc 1.0 Server with a threshold of 0.5 as a cutoff.
(TIF)

## References

[1] Thomashow MF. PLANT COLD ACCLIMATION: Freezing tolerance genes and regulatory mechanisms. Annu. Rev. Plant Physiol. Plant Mol. Biol. 6 1999;50: 571–99. 10.1146/annurev.arplant.50.1.571 15012220

[2] Uemura M, Tominaga Y, Nakagawara C, Shigematsu S, Minami A, Kawamura Y. Responses of the plasma membrane to low temperatures. Physiol. Plant. Januar 2006;126(1): 81–9.

[3] Koster KL, Lynch DV. Solute Accumulation and Compartmentation during the Cold Acclimation of Puma Rye. Plant Physiol. 1 1992; 98(1): 108–13. 16668599 10.1104/pp.98.1.108 PMC1080156

[4] Sasaki K, Imai R. Pleiotropic roles of cold shock domain proteins in plants. Front. Plant. Sci. 1 2002;2: 116. 10.3389/fpls.2011.00116 PMC3355641 22639630

[5] Sandve S, Rudi H, Asp T, Rognli O. Tracking the evolution of a cold stress associated gene family in cold tolerant grasses. BMC Evol. Biol. 9 2008;8(245). 10.1186/1471-2148-8-245 PMC2542378 18775065

[6] Zhang C, Zhang H, Wang L, Zhang J, Yao H. Purification of Antifreeze Protein from Wheat Bran (Triticum aestivum L.) Based on Its Hydrophilicity and Ice-binding Capacity. J. Agric. Food Chem. 9 2007;55(19): 7654–8. 10.1021/jf0715065 17715897

[7] Hon WC, Griffith M, Mlynarz A, Kwok YC, Yang D. Antifreeze proteins in winter rye are similar to pathogenesis-related proteins. Plant Physiol. 1995;109(3): 879–89. 8552719 10.1104/pp.109.3.879 PMC161389

[8] Pudney PD, Buckley SL, Sidebottom CM, Twigg SN, Sevilla MP, Holt CB, et al The physico-chemical characterization of a boiling stable antifreeze protein from a perennial grass (Lolium perenne). Arch. Biochem. Biophys. 2 2003;410(2): 238–45. 12573283 10.1016/s0003-9861(02)00697-5

[9] Smallwood M, Worrall D, Byass L, Elias L, Ashford D, Doucet CJ, et al Isolation and characterization of a novel antifreeze protein from carrot (Daucus carota). Biochem. J. 6 1999;340(Pt2): 385–91. 10333479 PMC1220261

[10] Raymond JA, DeVries AL. Adsorption inhibition as a mechanism of freezing resistance in polar fishes. Proc. Natl. Acad. Sci. U.S.A. 6 1977;74(6): 2589–93. 267952 10.1073/pnas.74.6.2589 PMC432219

[11] Knight CA, DeVries AL. Effects of polymeric, nonequilibrium “antifreeze” upon ice growth from water. J. Cryst. Growth. 10 1994;143(3–4): 301–10.

[12] Graham LA, Liou Y, Walker VK, Davies, PL. Hyperactive antifreeze protein from beetles. Nature. 8 1997;388: 727–8. 10.1038/41908 9285581

[13] Doucet D, Tyshenko MG, Kuiper MJ, Graether SP, Sykes BD, Daugulis AJ, et al Structure-function relationships in spruce budworm antifreeze protein revealed by isoform diversity. Eur. J. Biochem. 10 2000;267(19): 6082–8. 10998070 10.1046/j.1432-1327.2000.01694.x

[14] Griffith M, Ala P, Yang DS, Hon W.C., and Moffatt B.A. Antifreeze protein produced endogenously in winter rye leaves. Plant Physiol. 10 1992;100(2): 593–6. 16653033 10.1104/pp.100.2.593 PMC1075599

[15] Middleton AJ, Marshall CB, Faucher F, Bar-Dolev M, Braslavsky I, Campbell RL, et al Antifreeze protein from freeze-tolerant grass has a beta-roll fold with an irregularly structured ice-binding site. J. Mol. Biol. 3 2012;416(5): 713–24. 10.1016/j.jmb.2012.01.032 22306740

[16] Urrutia ME, Duman JG, Knight CA. Plant thermal hysteresis proteins. Biochim. Biophys. Acta. 5 1992;1121(1–2): 199–206. 1599942 10.1016/0167-4838(92)90355-h

[17] Huang T, Duman JG. Cloning and characterization of a thermal hysteresis (antifreeze) protein with DNA-binding activity from winter bittersweet nightshade, Solanum dulcamara. Plant Mol. Biol. 3 2002;48(4): 339–50. 11905961 10.1023/a:1014062714786

[18] Gupta R, and Deswal R. Refolding of β-Stranded Class I Chitinases of Hippophae rhamnoides Enhances the Antifreeze Activity during Cold Acclimation. PLoS ONE, 3 2014. 10.1371/journal.pone.0091723 PMC3953593 24626216

[19] Duman JG. Purification and characterization of a thermal hysteresis protein from a plant, the bittersweet nightshade Solanum dulcamara. Biochim. Biophy. Acta. 5 1994;1206(1): 129–35. 10.1016/0167-4838(94)90081-7 8186242

[20] Bredow M, Vanderbeld B, Walker VK. Ice-binding proteins confer freezing tolerance in transgenic Arabidopsis thaliana. Plant Biotech. J. 6 2016. 10.1111/pbi.12592 PMC5253476 27317906

[21] Khanna HK, Daggard GE. Targeted expression of redesigned and codon optimised synthetic gene leads to recrystallization inhibition and reduced electrolyte leakage in spring wheat at sub-zero temperatures. Plant Cell Rep. 12 2006;25(12): 1336–46. 10.1007/s00299-006-0191-9 16847628

[22] Zhang C, Fei SZ, Arora R, Hannapel DJ. Ice recrystallization inhibition proteins of perennial ryegrass enhance freezing tolerance. Planta. 6 2010;232(1); 155–64. 10.1007/s00425-010-1163-4 20379831

[23] Gong Z, Ewart KV, Hu Z, Fletcher GL, Hew CL. Skin Antifreeze Protein Genes from the Winter Flounder, Pleuronectes americanus, Encode Distinct and Active Polypeptides without the Secretory Signal and Pro Sequences. J. Biol. Chem. 2 1996;271(8): 4106–12. 8626748 10.1074/jbc.271.8.4106

[24] Scott GK, Hew CL, Davies PL. (1985) Antifreeze protein genes are tandemly linked and clustered in the genome of the winter flounder. Proc. Natl., Acad. Sci. U.S.A. 5 1985;9(issue): 2613–7. 10.1073/pnas.82.9.2613 PMC397614 3857603

[25] Doucet D, Tyshenko MG, Davies PL, Walker VK. A family of expressed antifreeze protein genes from the moth, Choristoneura fumiferana. Eur. J. Biochem. 1 2002;269(1): 38–46. 11784296 10.1046/j.0014-2956.2001.02628.x

[26] Colton-Gagnon K, Ali-Benali MA, Mayer BF, Dionne R, Bertrand A, Carmo SD, et al (2013) Comparative analysis of the cold acclimation and freezing tolerance capacities of seven diploid Brachypodium distachyon accessions. Ann. Bot. 3 2013;113(4): 681–93. 10.1093/aob/mct283 24323247 PMC3936580

[27] Li C, Rudi H, Stockinger EJ, Cheng H, Cao M, Fox SE, et al Comparative analyses reveal potential uses of *Brachypodium distachyon* as a model for cold stress responses in temperate grasses. BMC Plant Biol. 5 2012;12(65), 10.1186/1471-2229-12-65 PMC3487962 22569006

[28] Middleton AJ, Brown AM, Davies PL, Walker VK. Identification of the ice-binding face of a plant antifreeze protein. FEBS Lett. 2 2009;583(4): 815–9. 10.1016/j.febslet.2009.01.035 19185572

[29] Alves S, Worland B, Thole V, Snape J, Bevan M, Vain P. A protocol for Agrobacterium-mediated transformation of Brachypodium distachyon community standard line Bd21. Nature Prot. 2009;4(5): 638–49. 10.1038/nprot.2009.30 19360019

[30] Middleton AJ, Vanderbeld B, Bredow M, Tomalty H, Davies PL, Walker VK. Isolation and characterization of ice-binding proteins from higher plants. Methods Mol. Biol. 2014;1116: 255–77. 10.1007/978-1-4939-0844-8_19 24852641

[31] Fursova OV, Pogorelko GV, Tarasov VA. Identification of ICE2, a gene involved in cold acclimation which determines freezing tolerance in Arabidopsis thaliana. Gene. 1 2009;429(1–2): 98–103. 10.1016/j.gene.2008.10.016 19026725

[32] Vogel J, Hill T. High-efficiency Agrobacterium-mediated transformation of Brachypodium distachyon inbred line Bd21-3. Plant Cell Rep. 3 2008;27(3): 471–8. 10.1007/s00299-007-0472-y 17999063

[33] Hong SY, Seo PJ, Yang MS, Xiang F, Park CM. Exploring valid reference genes for gene expression studies in *Brachypodium distachyon* by real-time PCR. BMC Plant Biol. 11 2008;8(112):10.1186/1471-2229-8-112 PMC2588586 18992143

[34] Sandve SR, Kosmala A, Rudi H, Fjellheim S, Rapacz M, Yamada T, Rognli OA. Molecular mechanisms underlying frost tolerance in perennial grasses adapted to cold climates. Plant Sci. 1 2011;180(1): 69–77. 10.1016/j.plantsci.2010.07.011 21421349

[35] Hays LM, Feeney RE, Crowe LM, Crowe JH, Oliver AE. Antifreeze glycoproteins inhibit leakage from liposomes during thermotrophic phase transitions. Proc. Natl Acad. Sci. U.S.A. 6 1996;93(13): 6835–40. 8692905 10.1073/pnas.93.13.6835 PMC39114

[36] Rubinsky B, Arav A, Fletcher GL. Hypothermic protection: a fundamental property of “antifreeze” proteins. Biochem. Biophys. Res. Commun. 10 1991;180(2): 566–71. 1953726 10.1016/s0006-291x(05)81102-7

[37] Tomczak MM, Hincha DK, Estrada SD, Wolkers WF, Crowe LM, Feeney RE, et al A mechanism for stabilization of membranes at low temperatures by an antifreeze protein. Biophys. J. 2 2002;82(2): 874–81. 10.1016/S0006-3495(02)75449-0 11806929 PMC1301896

[38] Fei YB, Sun LH, Huang T, Shu NH, Gao SQ, Jian LC. Isolation and identification of antifreeze protein with high activity in Ammopiptanthus mongolicus (in Chinese with English Abstract). Acta Bot. Sin. 1994;36(8): 649–50.

[39] Gupta R, Deswal R. Low temperature stress modulated secretome analysis and purification of antifreeze protein from Hippophae rhamnoides, a Himalayan wonder plant. J. Proteome Res. 5 2012;11(5): 2684–96. 10.1021/pr200944z 22486727

[40] Lu CF, Jian LC, Kuang TY. Secretory antifreeze protein produced in suspension culture cells of Rhodiola algida var. tangutica during cold acclimation. Progress Biochem. Biophy. 10 2000;27: 555–9.

[41] Worrall D, Elias L, Ashford D, Smallwood M, Sidebottom C, Lillford P, et al A carrot leucine-rich-repeat protein that inhibits ice recrystallization. Science. 10 1998; 282(5386): 115–7. 9756474 10.1126/science.282.5386.115

[42] Kontogiorgos V, Regand A, Yada RY, Goff HD. Isolation and characterization of ice structuring proteins from cold acclimated winter wheat grass extract for recrystallization inhibition in frozen foods. J. Food Biochem. 4 2007;31(2): 139–60.

[43] Lauersen KJ, Middleton A, Davies PL, Walker VK. Expression and characterization of an antifreeze protein from the perennial rye grass, Lolium perenne. Cryobiology. 6 2011;62(3): 194–201. 10.1016/j.cryobiol.2011.03.003 21457707

[44] Yang DSC, Hon W, Bubanko S, Xue Y, Seetharaman J, Hew CL, Sicheri F. Identification of the Ice-Binding Surface on a Type III Antifreeze Protein with a “Flatness Function” Algorithm. Biophys. J. 5 1998;74(5): 2142–51. 10.1016/S0006-3495(98)77923-8 9591641 PMC1299557

[45] Zhang DQ, Liu B, Feng DR, He YM, Wang JF. Expression, purification and antifreeze activity of carrot antifreeze protein and its mutants. Protein Express. Purif. 6 2004;35(2): 257–63. 10.1016/j.pep.2004.01.019 15135400

[46] Griffith M, Yaish MW. Antifreeze proteins in overwintering plants: a tale of two activities. Trends Plant Sci. 8 2004;9(8): 399–405. 10.1016/j.tplants.2004.06.007 15358271

[47] Burcham TS, Knauf MJ, Osuga DT, Feeney RE, Yeh Y. Antifreeze glycoproteins influence of polymer length and ice crystal habit on activity. Biopolymers. 7 1984;23(7): 1379–95. 10.1002/bip.360230720 6466773

[48] Nishimiya Y, Sato R, Takamichi M, Miura A, Tsuda S. Co-operative effect of the isoforms of type III antifreeze protein expressed in Notched-fin eelpout, Zoarces elongates. FEBS J. 1 2005;272(2): 482–92. 10.1111/j.1742-4658.2004.04490.x 15654886

[49] Yu S, Pilot G. Testing the efficiency of plant artificial microRNAs by transient expression in NIcotiana benthamiana reveals additional action at the translational level. Front. Plant Sci. 11 2014; 5:622 10.3389/fpls.2014.00622 25477887 PMC4237044

[50] Iwakawa HO, Tomari Y. Molecular insights into microRNA-mediated translational repression in plants. Mol. Cell. 11 2013;52(4): 591–691. 10.1016/j.molcel.2013.10.033 24267452

[51] Brodersen P, Sakvarelidze-Achard L, Bruun-Rasmussen M, Dunoyer P, Yamamoto YY, Sieburth K, Voinnet O. Widespread translational inhibition by plant miRNAs and siRNAs. Science. 5 2008; 320(5880): 1185–90. 10.1126/science.1159151 18483398

[52] Antikainen M, Griffith M. Antifreeze protein accumulation in freezing-tolerant cereals. Physiol. Plant. 3 1997;99(3): 423–32.

[53] Marentes E, Griffith M, Mlynarz A, Brush RE. Proteins accumulate in the apoplast of winter rye leaves during cold acclimation. Physiol. Plant. 4 1993;87(4): 499–507.

[54] Bajji M, Kinet J, Lutts S. The use of the electrolyte leakage method for assessing cell membrane stability as a water stress tolerance test in durum wheat. Plant Growth Regul. 1 2002;36(1): 61–70.

[55] Hirano Y, Nishimiya Y, Matsumoto S, Matsushita M, Todo S, Miura A, et al Hypothermic preservation effect on mammalian cells of type III antifreeze proteins from notched-fin eelpout. Cryobiology. 8 2008;57(1): 46–51. 10.1016/j.cryobiol.2008.05.006 18603237

[56] Muthukumaran J, Manivel P, Kannan M, Jeyakanthan J, Krishna R. A framework for classification of antifreeze proteins in over wintering plants based on their sequence and structural features. J. Bioinform. Seq. Anal. 2011;3: 70–88.

[57] Albrecht C, Russinova E, Hecht V, Baaijens E, de Vries S. The *Arabidopsis thaliana* SOMATIC EMBRYOGENESIS RECEPTOR-LIKE KINASES1 and 2 control male sporogenesis. Plant Cell. 12 2005;17(12): 3337–49. 10.1105/tpc.105.036814 16284305 PMC1315373

[58] Clark SE, Williams RW, Meyerowitz EM. The *CLAVATA1* gene encodes a putative receptor kinase that controls shoot and floral meristem size in Arabidopsis. Cell. 5 1997;89(4): 575–85. 9160749 10.1016/s0092-8674(00)80239-1

[59] Colcombet J, Boisson-Dernier A, Ros-Palau R, Vera CE, Schroeder JI. (2005) Arabidopsis SOMATIC EMBRYOGENESIS RECEPTOR KINASES1 and 2 are essential for tapetum development and microspore maturation. Plant Cell. 12 2005;17(12): 3350–61. 10.1105/tpc.105.036731 16284306 PMC1315374

[60] Gao M, Wang X, Wang D, Xu F, Ding X, Zhang Z, et al Regulation of cell death and innate immunity by two receptor-like kinases in Arabidopsis. Cell Host Microbe. 6 2009;6(1): 34–44. 10.1016/j.chom.2009.05.019 19616764

[61] Go´mez-Go´mez L, Bauer Z, Boller T. Both the extracellular leucine-rich repeat domain and the kinase activity of FSL2 are required for flagellin binding and signaling in Arabidopsis. Plant Cell. My 2001;13(5): 1155–63. PMC135565 11340188

[62] Jinn TL, Stone JM, Walker JC. HAESA, an Arabidopsis leucine-rich repeat receptor kinase, controls floral organ abscission. Gene Dev. 1 2000;14(1): 108–17. 10640280 PMC316334

[63] Torii KU, Mitsukawa N, Oosumi T, Matsuura Y, Yokoyama R, Whittier RF, et al The Arabidopsis *ERECTA* gene encodes a putative receptor protein kinase with extracellular leucine-rich repeats. Plant Cell. 4 1996;8(4): 735–46. 10.1105/tpc.8.4.735 8624444 PMC161133

[64] Zhao DZ, Wang GF, Speal B, Ma H. The excess microsporocytes1 gene encodes a putative leucine-rich repeat receptor protein kinase that controls somatic and reproductive cell fates in the Arabidopsis anther. Gene Dev. 8 2002;16(15): 2021–31. 10.1101/gad.997902 12154130 PMC186413

[65] Schreiber AW, Sutton T, Caldo RA, Kalashyan E, Lovell B, Mayo G, et al Comparative transcriptomics in the Triticeae. BMC Genomics. 6 2009. 10.1186/1471-2164-10-285 PMC2717122 19558723

[66] Yu Y, Guo G, Lv D, Hu Y, Li J, Li X, et al (2014) Transcriptome analysis during seed germination of elite Chinese bread wheat cultivar Jimai 20. BMC Plant Biol. 1 2014. 10.1186/1471-2229-14-20 PMC3923396 24410729

