# Supplementary figures and images for: Knockdown of Ice-Binding Proteins in Brachypodium distachyon Demonstrates Their Role in Freeze Protection
Source: PLoS One. 2016 Dec 13;11(12):e0167941. doi: 10.1371/journal.pone.0167941 (PMC5154533; doi:10.1371/journal.pone.0167941)

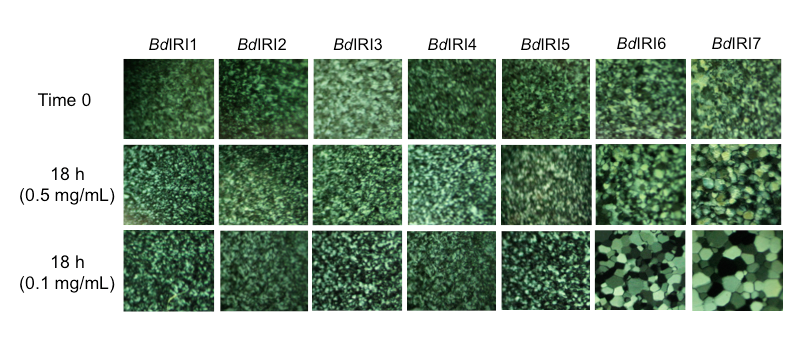

Supplement: S1 Fig — A dilution series was conducted on purified BdIRI proteins and ice crystals were observed after annealing at −4°C for 18h. Assay was conducted in triplicate. (TIF) [file pone.0167941.s001.tif]

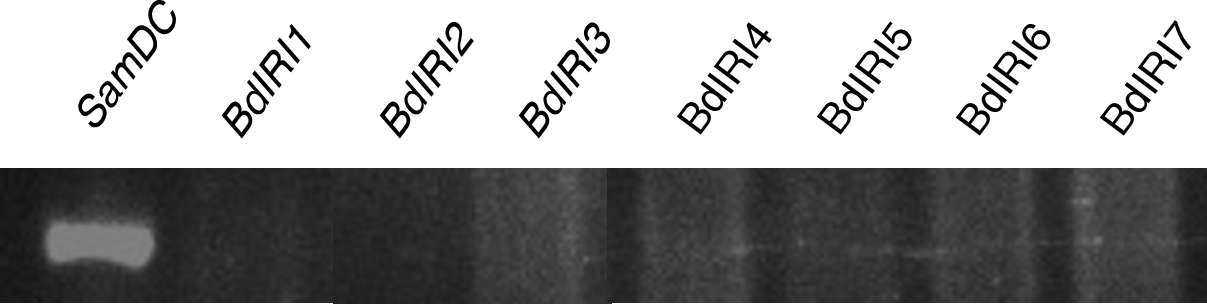

Supplement: S2 Fig — Transcripts were amplified for each BdIRI using sequence specific primers (Table 1). SamDC served as a PCR loading reference. Assays were conducted in duplicate with identical results. (TIF) [file pone.0167941.s002.tif]

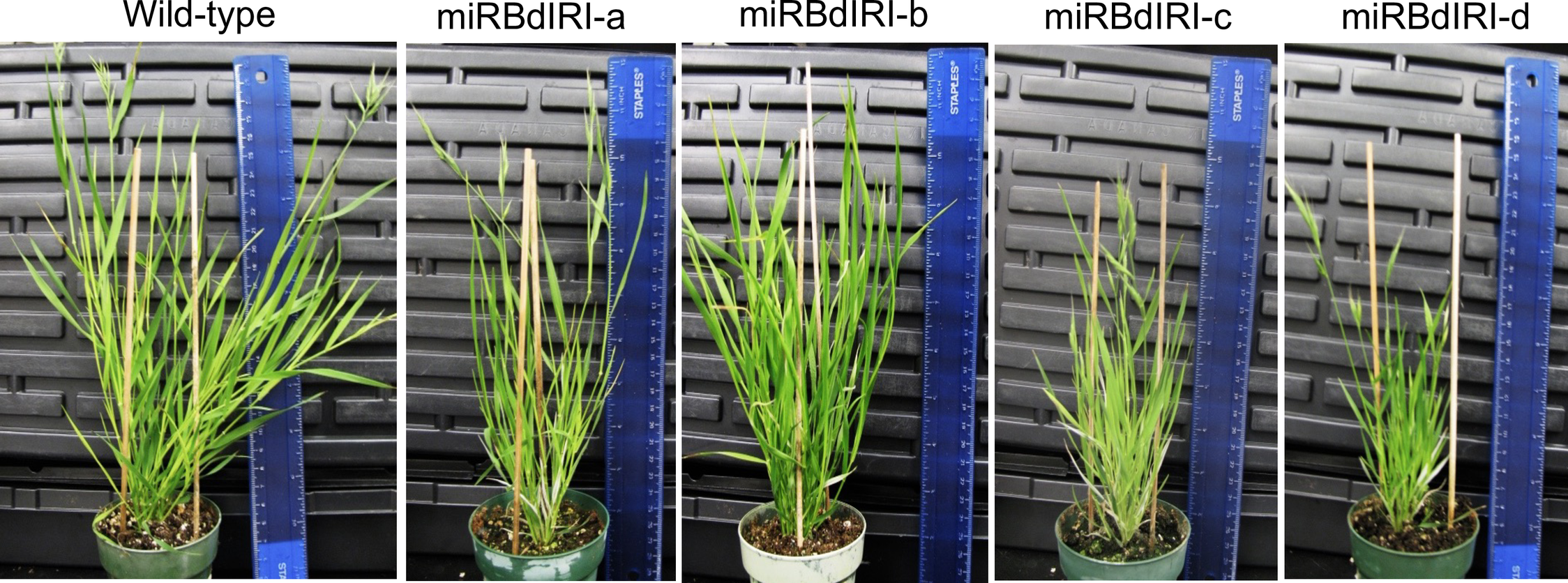

Supplement: S3 Fig — Twelve-week-old transgenic BdIRI knockdown lines show shorter stature and less above ground biomass compared to wild-type B. distachyon plants. (TIF) [file pone.0167941.s003.tif]

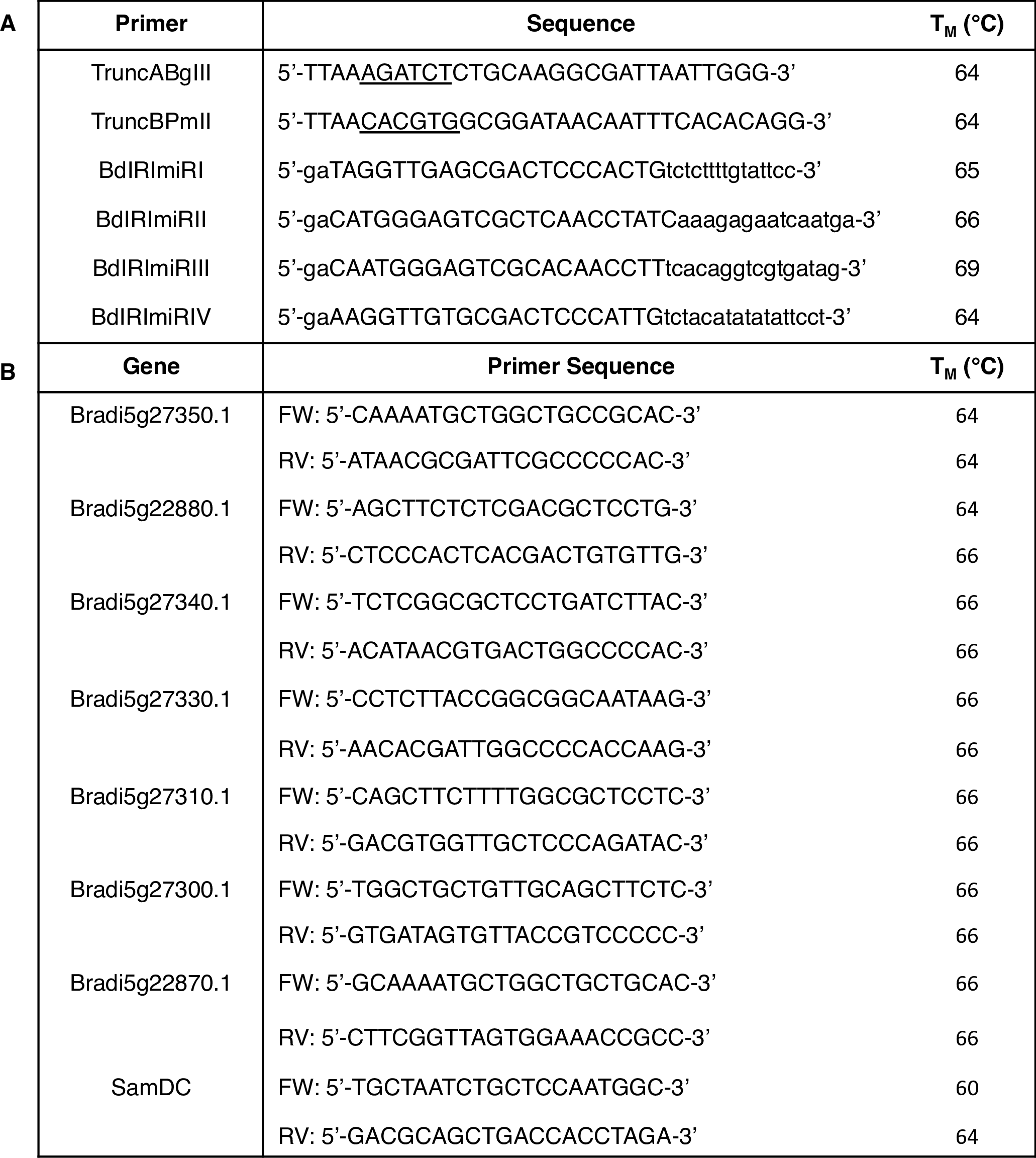

Supplement: S1 Table — The primers used for the generation of the miRBdIRI construct (A) and the forward (FW) and the reverse (RV) primers used for reverse-transcription PCR of BdIRI transcripts (B) are shown. Restriction sites are underlined and melting temperatures (TM) are indicated. (TIF) [file pone.0167941.s004.tif]

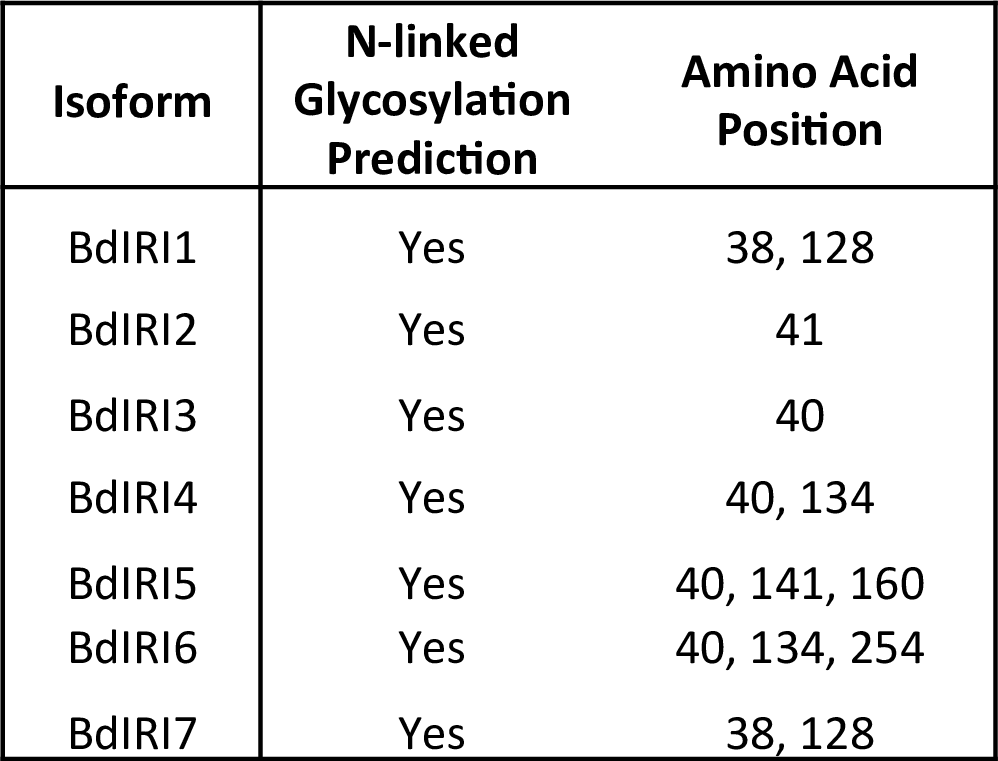

Supplement: S2 Table — Predictions using BdIRI1-7 sequences were made using the NetNGlyc 1.0 Server with a threshold of 0.5 as a cutoff. (TIF) [file pone.0167941.s005.tif]
